# Characterization of the mitochondrial genome of the Qilian yak (*Bos grunniens*) with a phylogenetic analysis of the family Bovidae (Artiodactyla)

**DOI:** 10.1080/23802359.2019.1644553

**Published:** 2019-07-22

**Authors:** Xian Guo, Xiaoyun Wu, Pengjia Bao, Suonan Zhao, Xita Za, Chunnian Liang, Min Chu, Jie Pei, Ping Yan

**Affiliations:** a Key Laboratory of Yak Breeding Engineering of Gansu Province, Lanzhou Institute of Husbandry and Pharmaceutical Sciences, Chinese Academy of Agricultural Sciences, Lanzhou, People’s Republic of China;; b Institute of Animal Husbandry and Veterinary Medicine, Haibei Tibetan Autonomous Prefecture, Xihai, People’s Republic of China;; c Animal Husbandry and Veterinary Station of Qilian County, Qilian, People’s Republic of China

**Keywords:** Bayesian inference, domestic yak, mitochondrial genome, phylogenetic analysis, polyphyly

## Abstract

Qilian yak (*Bos grunniens*) is a local breed of yak with high adaptation to the high-elevation, cold, and anoxic environments. In this study, its complete mitochondrial genome was assembled from high-throughput sequencing reads. The mitogenome is 16,324 bp long with an A + T-biased nucleotide composition and has the typical set of 37 animal mitochondrial genes and the noncoding control region. Phylogenetic analysis supported the monophyly of the subfamilies Aepycerotinae, Alcelaphinae, Bovinae, Caprinae, and Cephalophinae, confirmed the polyphyly of the family Antilopinae within the family Bovidae, and indicated the close relatedness between domestic yaks (*Bos grunniens*) and wild yaks (*Bos mutus*).

Domestic yaks (*Bos grunniens*) are widely maintained in China and many parts of central Asia (Leslie and Schaller 2009), and many local breeds have been developed during the long history of domestication (Qiu et al. 2012). Qilian yak is a yak breed locally maintained in Qilian County, Haibei Tibetan Autonomous Prefecture, Qinghai Province, China and is highly adapted to the high-elevation, cold, and anoxic environments. In this study, its complete mitochondrial genome was assembled from high-throughput sequencing reads and is currently available from GenBank under the accession number MK922355.

A blood sample of Qilian yak was collected from Qilian County (Qinghai Province, China; 38°66′N, 99°25′E) and was used for genomic DNA extraction with the QIAamp DNA Blood Mini Kit (Qiagen, CA, USA). A voucher specimen is deposited in the Key Laboratory of Yak Breeding Engineering of Gansu Province, Lanzhou Institute of Husbandry and Pharmaceutical Sciences (Lanzhou, Gansu Province, China). The genomic DNA coded as NO.20190419, which was extracted from Qilian yak, is stored at −80 °C (ultra-deep-freeze refrigerator) in the sample storage room of our department. High-throughput DNA sequencing was carried out with the Illumina HiSeq X™ Ten Sequencing System (Illumina, CA, USA) by Annoroad Gene Technology (Beijing, China). Totally, 14.2 M raw reads of 150 bp were obtained and used for the mitogenome assembly with the program MITObim v1.9 (Hahn et al. 2013); the reference sequence (JQ692071) was previously published by Qiu et al. (2012). Genomic annotation was conducted in Geneious R11 (Biomatters Ltd., Auckland, New Zealand) by aligning with those of its congeners and by using the MITOS web server (Bernt et al. 2013).

The mitochondrial genome of Qilian yak is 16,324 bp long with an A + T-biased nucleotide composition and harbors the typical set of 37 animal mitochondrial genes (13 protein-coding genes/PCGs, 22 tRNAs, and two rRNAs) and one non-coding control region. The PCGs are initiated with the typical ATA or ATG codons and are terminated with TAA, TAG or the incomplete stop codon T––. The 22 tRNAs range in length from 60 (*tRNA-Ser^AGN^*) to 75 bp (*tRNA-Leu^UUR^*). The two rRNAs, which are, separated by *tRNA-Val*, 957 bp (*12S rRNA*) and 1571 bp (*16S rRNA*) in length, respectively. The control region is 894 bp long with an A + T content of 60.6% and is located between *tRNA-Pro* and *tRNA-Phe*.

A Bayesian phylogeny was reconstructed for a total of 58 species within the family Bovidae using all 13 protein-coding genes with MrBayes v3.1.1 (Ronquist and Huelsenbeck 2003) as implemented in TOPALi v2.5 (Milne et al. 2009) (Figure 1). Phylogenetic analysis revealed close relatedness between domestic yak (as represented by the Qilian yak) and the wild yak (*Bos mutus*; KY829451) (Wu et al. 2018) with a pairwise sequence identity of 99.9%, which could be explained by the long history of hybridization between these two species (Leslie and Schaller 2009; Wang et al. 2016). Besides, our analysis lends support to the monophyly of the subfamilies Aepycerotinae, Alcelaphinae, Bovinae, Caprinae, and Cephalophinae but confirms the polyphyly of Antilopinae as previously reported (Matthee and Davis 2001). Furthermore, it also supports the proposed inclusion of the genus *Pelea* within the subfamily Reduncinae (Gatesy et al. 1997; Hassanin and Douzery 1999).

**Figure 1.**
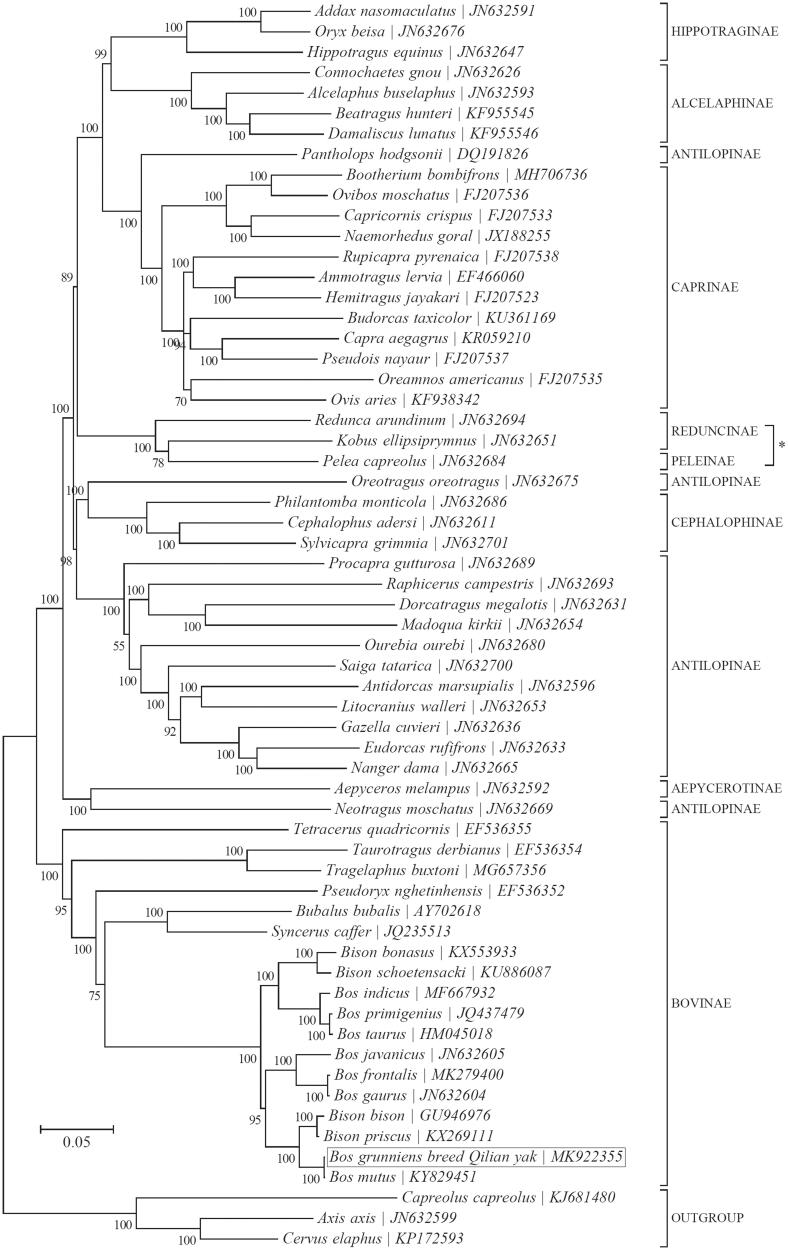
Phylogeny of the family Bovidae based on the Bayesian analysis of the concatenated sequences of 13 mitochondrial protein-coding genes (alignment size: 11,067 bp). The best-fit nucleotide substitution model is ‘GTR + G+I’. The support values are placed next to the nodes. Three species within the family Cervidae were included as outgroup taxa. Subfamily-level taxonomy was retrieved from GenBank-Taxonomy database and was shown for each taxon.
